# Nrf2-driven CD36 and HO-1 gene expression in circulating monocytes correlates with favourable clinical outcome in pregnancy-associated malaria

**DOI:** 10.1186/s12936-015-0888-8

**Published:** 2015-09-18

**Authors:** Agnès Aubouy, David Olagnier, Gwladys Bertin, Sem Ezinmegnon, Clarisse Majorel, Saliha Mimar, Achille Massougbodji, Philippe Deloron, Bernard Pipy, Agnès Coste

**Affiliations:** Institut de Recherche pour le Développement (IRD), Université Paul Sabatier Toulouse III, UMR 152 Pharma-Dev, CHU Rangueil, Bâtiment L1, 1 Avenue du Pr Jean Poulhès, 31059 Toulouse, France; Institut de Recherche pour le Développement (IRD), PRES Sorbonne Paris Cité, Université Paris Descartes, UMR 216 Mère et enfant face aux infections tropicales, Paris, France; Centre d’Etude et de Recherche sur le Paludisme Associé à la Grossesse et l’Enfance (CERPAGE), Cotonou, Benin; Lady Davis Institute-Jewish General Hospital, McGill University, Montreal, Canada; Laboratoire Insulaire du Vivant et de l’Environnement (LIVE-EA 4243), Université de la Nouvelle-Caledonie (UNC), Nouméa, New Caledonia

**Keywords:** Pregnancy-associated malaria, Clinical outcomes, Monocytes, CD36, Nrf2, HO-1

## Abstract

**Background:**

Pregnancy-associated malaria (PAM) constitutes one of the most severe forms of malaria infection leading to fetal growth restriction and high risk of infant death. The severity of the pathology is largely attributed to the recruitment of monocytes and macrophages in the placenta which is evidenced by dysregulated inflammation found in placental blood. Importantly, CD36^+^ monocytes/macrophages are also thought to participate in the tight control of the pro- and anti-inflammatory responses following *Plasmodium* detection through elimination of apoptotic cells and malaria-infected erythrocytes, internalization and recycling of oxidized forms of low-density lipoprotein and collaboration with TLR2 in pro-inflammatory response. Interestingly, previous work demonstrated that CD36 expression was upregulated on inflammatory macrophages following stimulation of the Nrf2 transcription factor, whilst the PPARγ pathway was inhibited and non-functional in the same inflammatory conditions. This current study examined the possible role of Nrf2-driven gene expression, CD36 and Haem-Oxygenase-1 (HO-1), in PAM clinical outcomes.

**Methods:**

Clinical data and biological samples including peripheral blood mononuclear cells were collected from 27 women presenting PAM. Polychromatic flow cytometry was used to characterize innate immune cell subpopulations and quantify CD36 protein expression level on monocytes. mRNA levels of CD36, PPARγ, Nrf2 and HO-1 were determined by qPCR and related to clinical outcomes. Finally, the capacity of monocytes to modulate CD36 expression upon rosiglitazone or sulforaphane treatment, two respective PPARγ or Nrf2 activators, was also investigated.

**Results:**

The CD36 receptor, mostly expressed by CD14^+^ circulating monocytes, statistically correlated with increased infant birth weights. Interestingly, mRNA levels of the transcription factor Nrf2 and the enzyme HO-1 also correlated with lower parasitaemia and increased infant birth weight, while PPARγ mRNA levels did not. Finally, monocytes isolated from low infant birth weight pregnant women were capable of up-regulating CD36 via the Nrf2 pathway ex vivo.

**Conclusions:**

Altogether these results suggest that Nrf2-driven CD36 and HO-1 expression on innate immune cells could contribute to a protective and detoxifying mechanism during PAM. More powered and mechanistical studies are however needed to strengthen the conclusions of this study.

**Electronic supplementary material:**

The online version of this article (doi:10.1186/s12936-015-0888-8) contains supplementary material, which is available to authorized users.

## Background

Each year, more than 125 million pregnant women are at risk of pregnancy-associated malaria (PAM), a severe form of malaria infection that affects both mothers and infants, causing maternal anaemia, pre-term delivery, fetal growth restriction and a higher risk of infant death [1, 2]. The severity of the pathology is attributed to the sequestration of *Plasmodium falciparum*-infected erythrocytes (*Pf*-iE) in placental intervillous blood spaces that leads to the recruitment of monocytes and macrophages to the placenta [3, 4]. Several studies have demonstrated a link between monocyte/macrophage recruitment and adverse outcomes such as low birth weight and maternal anaemia, but also functional damage to placental villi and disturbances to feto-maternal exchanges [4–7]. The mechanisms proposed imply monocyte/macrophage activation which is evidenced by high levels of pro-inflammatory cytokines in placental blood [8–11].

On the other hand, it is largely accepted that monocyte and macrophage infiltrates might also have a beneficial role in defence mechanisms, such as non-opsonic CD36-dependent phagocytosis, antibody-dependent cellular inhibition (ADCI), opsonic phagocytosis and the production of cytokines that direct both cellular and humoral immunity [12, 13]. CD36, a membrane glycoprotein present on many mammalian cells types, including monocyte/macrophages, is a multifaceted receptor that displays an impressive range of functions [reviewed in 14]. On monocyte/macrophages, CD36 acts as a scavenger receptor to protect the host from inflammation through phagocytosis of apoptotic cells (efferocytosis) and diverse pathogens including *Pf*-iE. The initial sensing of the invading malaria parasite is mediated through recognition of highly conserved microbial structures by pattern-recognition receptors (PRRs) expressed on monocytes and macrophages [12, 15]. The CD36 scavenger receptor, a PRR, is known for its involvement in recognizing and internalizing non-opsonized *Pf*-iE, in a non pro-inflammatory manner. Indeed, macrophages from CD36 knock-out mice were shown to engulf significantly less iEs than macrophages from CD36 wild-type mice, and displayed earlier peaks of parasitaemia, higher parasite densities and mortality rates [16–18]. CD36 also contributes in the internalization and the recycling of oxidized forms of low-density lipoprotein (oxLDL) [14]. In complement with its phagocytic activities, CD36 is also known to contribute to the regulation of the inflammatory response through cooperation with TLR2 [19]. CD36 and TLR2 collaboration leads to a pro-inflammatory response via ERK, p38, MAPK, JNK and NF-kB signaling following interaction with malaria-GPI anchors [19, 20]. On endothelial cells, CD36 is known for its role as a negative regulator of angiogenesis and has also been identified as a sequestration receptor for *Pf*-iE during malaria [21], although the main sequestration receptor during PAM is chondroitin sulfate A (CSA) [22]. Even though *P. falciparum* parasites infecting pregnant women are known to predominantly display CSA-adherent phenotype and low adhesion to CD36 [23], the multifaceted roles of CD36 have prompted us to examine the possible involvement that this receptor may play in PAM clinical outcomes.

CD36 expression is under the transcriptional control of the peroxisome proliferator-activated receptor gamma (PPARγ) and the nuclear factor erythroid-derived 2-like 2 (NF-E2L2 or Nrf2) [24, 25]. During malaria blood stage infection, haemoglobin metabolism by the parasite leads to reactive oxygen species (ROS) production that activates the transcription factors PPARγ and Nrf2 [26–28]. Importantly, it was demonstrated in vitro that human and murine macrophages downregulated CD36 expression in the presence of TNF, due to a failure to express and activate PPARγ [29]. However, unlike PPARγ agonists, Nrf2 activators maintained their capacity to enhance CD36 expression and CD36-mediated phagocytosis in inflammatory conditions [29]. During PAM, elevated pro-inflammatory cytokines are consistently found, particularly TNF, IFNγ and MCP-1 [8, 9], suggesting a possible impairment of the PPARγ pathway. Furthermore, the activation of the nuclear factor Nrf2 constitutes a key mechanism in the antioxidant defence response used by host cells during infection [30]. Among the hundreds of Nrf2-regulated genes, the enzyme haem oxygenase-1 (HO-1) is certainly one of the most important and was identified as a crucial detoxifier of free haem during malaria infection [31]. The role of HO-1 during malaria is however controversial and requires a better understanding. The protective role of HO-1 and carbon monoxide against cerebral malaria development in mice was previously demonstrated [32, 33], whereas other groups reported that high HO-1 was a risk factor for severe or cerebral malaria in African children [34, 35]. The detoxifying function of HO-1 is also closely related to another scavenger receptor, CD163, that binds the haptoglobin–haemoglobin complex, removes it from circulation by endocytosis, allowing subsequent degradation of haem by HO-1 [36]. Few reports have investigated CD163 as a biomarker during malaria, although it may constitute an important factor implied in the anti-inflammatory response.

To date, no study has demonstrated that monocytes/macrophages can contribute to malaria protection during pregnancy. Here, monocytes sampled from women presenting PAM at delivery in Benin (West Africa) were studied. The objective was to examine whether the expression of both CD36 and HO-1 by monocytes plays a role in clinical outcomes during PAM, based on the following hypothesis: (1) the scavenger receptor CD36 may be involved in the pro- and anti-inflammatory response, and to a lesser extent the elimination of *Pf*-iE during PAM; and (2) in a PAM context, the Nrf2 pathway may play an important role, both for its maintenance of CD36 expression and for its capacity to modulate the expression of detoxifying enzymes including HO-1. This study demonstrates that CD36 expression is directly correlated to higher infant birth weights. Furthermore, the importance of the Nrf2 pathway was also shown by an inverse relationship between Nrf2 mRNA levels and parasitaemia, and a positive correlation between HO-1 mRNA and a higher infant birth weight. Finally, CD36 expression on monocytes from mothers with low birth weights infants could be increased following Nrf2 stimulation, but not PPARγ. Altogether these results suggest that Nrf2-driven CD36 and HO-1 expression on innate immune cells could contribute to a protective and detoxifying mechanism during PAM.

## Methods

### Patients and ethical statement

Patient enrolment and ethical statement are described elsewhere [37]. Briefly, ethical clearance was obtained from the Institutional Ethics Committee of the Faculté des Sciences de la Santé at the Abomey Calavi University in Benin. Pregnant women were enrolled in Cotonou, southern Benin, in the Mother and Child Hospital (Hôpital de la Mère et de l’Enfant Lagune). Inclusion criterion was a positive diagnosis for *P. falciparum* infection at delivery. Parasitaemia was retrospectively quantified through Giemsa-stained thick blood smears prepared from peripheral blood and through placental impression smears.

### Clinical outcomes

Two clinical outcomes were studied: parasitaemia and infant birth weight. Both placental and peripheral parasitaemia were analysed, respectively named “P” and “pP”. As 1 % parasitaemia and 2500 g for infant birth weight (BW) are standard thresholds in clinical malaria studies, both thresholds were considered (P or pP >1 % and BW ≤2500 g) to define adverse outcomes.

### Blood sampling and PBMC isolation

Peripheral venous blood was collected from all patients in a vacutainer tube containing EDTA and processed within 4 h. Plasma was sampled after centrifugation and stored at −20 °C. Human peripheral blood mononuclear cells (PBMC) were isolated by a density gradient centrifugation method on Lymphoprep (Abcys, France).

### TNF and IL-10 plasma titration

The levels of TNF and IL-10 in plasma samples were determined using a commercially available OptiEIA kit (BD Biosciences) as per manufacturer’s instructions.

### Flow cytometric analysis

**Fig. 1:**
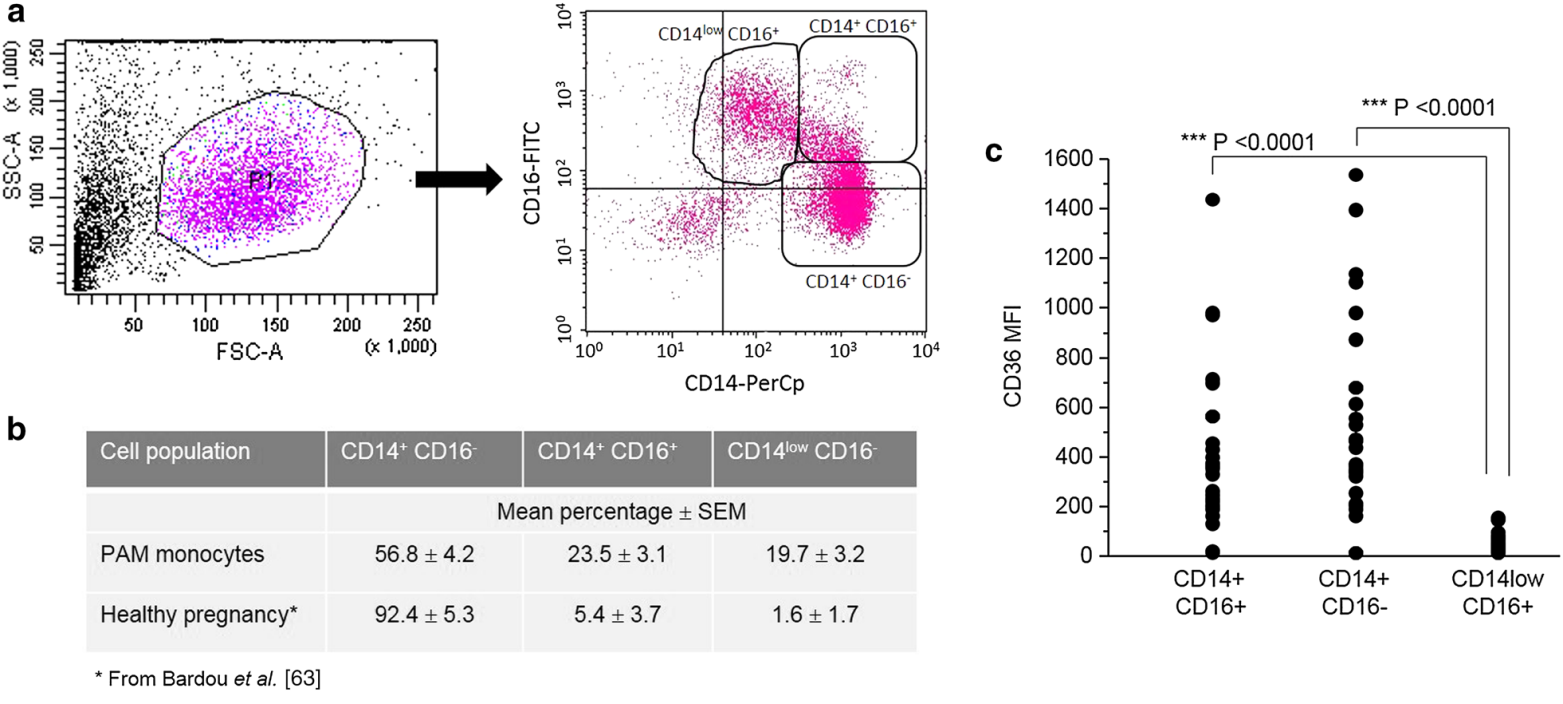
Relations between PAM, monocyte sub-populations and CD36 expression. **a** Gating strategy for the identification of monocyte sub-populations. Forward and side scattering parameters (FSC and SSC) were first used to gate monocytes among other leucocytes. Monocytes were sub-divided into CD14^+^ CD16^−^/CD14^+^ CD16^+^/CD14^low^ CD16^+^, according to CD14 and CD16 staining characteristics. Plots shown are representative examples. **b** Percentages of each monocyte sub-set found in PAM monocytes. **c** CD36 protein expression according to monocyte sub-populations in PAM. Geometric mean fluorescence intensity (MFI) was measured by flow cytometry for each monocyte sub-set. Values were compared by the Mann–Whitney U-test

Peripheral blood mononuclear cells were stained with CD14-PerCp, CD16-FITC (Immunotech), and CD36-APC (BD Pharmingen) antibodies during 15 min in the dark for monocyte identification. A minimum of 5000 cells were analysed for each data point. Acquisitions were performed on a FACScalibur using CellQuestPro software (Becton Dickinson, France). Forward and side scattering parameters (FSC and SSC) were first used to gate on monocytes among other leucocytes. Three CD14 CD16 monocytes subsets were defined according to anti-CD14 and anti-CD16 labeling (see Fig. 1).

### Monocyte activation and mRNA expression measurements by RT-qPCR

Monocytes were isolated by adherence to plastic for 1 h 30 min in M-SFM (Gibco Invitrogen) at 37 °C 5 % CO_2_. After several washes in pre-warmed PBS, monocytes were incubated during four additional hours in separate wells with 1 µM rosiglitazone (Cayman Chemical), 1 µM sulforaphane (Sigma Aldrich), or with medium alone (control wells). Cells were prepared for RNA extraction with the RNAqueous^®^-Micro Kit (Ambion, Life Technologies), as described by the supplier. Synthesis of cDNA was performed from a minimum of 100 ng of total RNA with Verso cDNA Synthesis Kit (Thermo Scientific) according to the manufacturer’s recommendations and primed with anchored oligo-dT. Quantitative real-time PCR was performed on a LightCycler 480 system using LightCycler 480 SYBR Green I Master (Roche Diagnostics). Serially diluted samples of pooled cDNA were used as external standards in each run for the quantification and results were expressed in fold induction relative to the respective control. The amplifications were performed for 60 cycles (10 s at 95 °C and 60 s at 60 °C) but the numbers of cycles needed for amplification (Cp) for all the genes tested was between 15 (18S) and 35 (PPARγ). The primers (at a final concentration of 10 mM) were designed using Primer 3 software, and are listed in Additional file 1: Table S1. *Human 18s* mRNA was used as the invariant control.

### Statistical analysis

All statistical analyses were carried out with non-parametric tests and performed with the software Statview. The Wilcoxon rank test was used to compare the distribution of continuous values. Values were compared according to categorized variables (birth weight, parasitaemia, clinical outcome, plasma TNF and IL-10 levels, CD36 protein expression level) by Mann–Whitney U-test, two-factor covariance analysis (ANOVA) and Kruskal–Wallis test. The Spearman correlation test was used to test the relation between continuous variables.

## Results

### Clinical, parasitological and biological data

Table 1 represents general clinical and parasitological data obtained from the pregnant women included in the study. Low birth weight was neither associated with age, gravidity, parasitaemia, nor cytokine levels. Women that displayed a peripheral parasitaemia ≥1 % were younger with a lower gravidity, higher prevalence of placental parasitaemia and increased IL-10 plasma levels. Placental parasitaemia was overall higher than peripheral parasitaemia (Wilcoxon rank test, P = 0.02). No adverse outcome was reported in the group of pregnant women following delivery, apart from the low birth weight.

**Table 1:** Clinical, parasitological and plasma cytokine levels of pregnant women

	All (N = 25)	BW ≤2.5 kg (N = 5)	P ≥1 % (N = 8)	pP ≥1 % (N = 10)
Age (years)	25 (22–30)	25 (21–37)	22 (20–23)**	23 (22–27)
Gravidity	2.0 (2.0–3.5)	2.0 (2.0–6.0)	2.0 (1.0–2.0)*	2.0 (1.0–3.0)
Birth weight (kg)	2.8 (2.5–3.1)	2.0 (1.7–2.2)**	2.8 (2.6–3.2)	2.9 (2.8–3.4)
P (%)	0.05 (0.01–3.5)	0.03 (0.02–2.9)	6.0 (4.1–10.8)***	2.2 (0.03–8.7)
pP (%)	1.0 (0.07–13.5)	0.1 (0.06–12.4)	28.0 (3.7–59.0)*	21.5 (10.0–63.0)***
TNF (pg/mL)	0 (0–0.8)	0 (0–12.2)	0 (0–0.1)	0 (0–0.6)
IL-10 (pg/mL)	0 (0–186)	111 (14–226)	628 (506–891)***	308 (12–732)

Data are presented in medians (interquartile range)

Data were compared according to the clinical criteria defined (BW ≤2.5 kg versus BW >2.5 kg, P <1 % versus P ≥1 %, pP <1 % versus pP ≥1 %) by Mann–Whitney U-test

*P* peripheral parasitaemia, *pP* placental parasitaemia

* *P* < 0.05, ** *P* < 0.005, *** *P* < 0.0005

### CD14 CD16 monocyte sub-populations differ by their levels of CD36 protein expression

Human monocytes were classified based on CD14 and CD16 expression in a major classical sub-population (CD14^+^ CD16^−^), and two minor sub-populations, intermediate (CD14^+^ CD16^+^) and non-classical monocytes (CD14^low^ CD16^+^) [38]. Polychromatic flow cytometry analysis was used to precisely identify the monocyte subsets based on CD14 and CD16, and determine their expression of CD36 (Fig. 1a). The mean percentages (±SEM) of each monocyte subset was 56.8 % (±4.2) CD14^+^ CD16^−^, 23.5 % (±3.1) CD14^+^ CD16^+^ and 19.7 % (±3.2) CD14^low^ CD16^+^ (Fig. 1b). As shown in Fig. 1c, CD36 protein expression was similar between the CD14^+^ CD16^−^ and CD14^+^ CD16^+^ subsets but significantly reduced in CD14^low^CD16^+^ monocytes (Mann–Whitney U-test, P < 0.0001).

### CD36 mRNA and protein expression levels correlate with increased birth weight

**Fig. 2:**
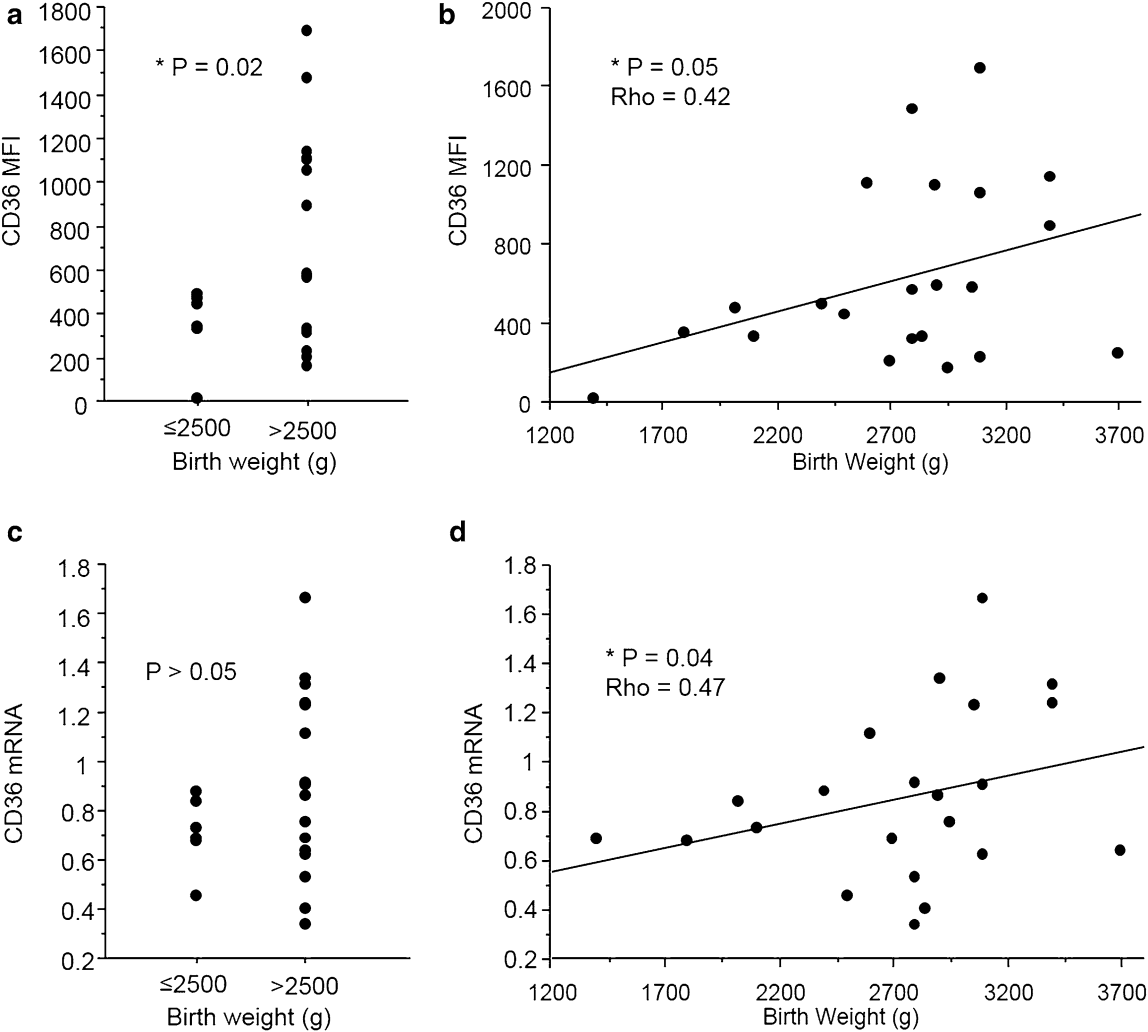
Relation between CD36 protein and mRNA expression and birth weight in PAM. **a**, **c** CD36 MFI and mRNA expression levels were compared according to birth weight threshold of 2.5 kg, by the two-factor covariance analysis (ANOVA). **b**, **d** CD36 MFI and mRNA expression levels were positively related to birth weight values by the Spearman correlation test

To evaluate the implication of the CD36 receptor on circulating monocytes during PAM, the relationship between clinical parameters and its expression level was analysed. As the number of pregnant women included in the study was relatively low (n = 27), it reduces the power of the statistical tests used. Thus, two complementary statistical approaches were used to consolidate the results, the two-factor covariance analysis (ANOVA) and the Spearman correlation test (see Fig. 2). High CD36 protein expression levels statistically correlated with increased birth weight according to both tests (Fig. 2a, b, P ≤ 0.05). CD36 mRNA levels also positively correlated with infant birth weight by the Spearman test (Fig. 2d, P < 0.05, Fig. 2c, P > 0.05). No other significant relationships were found with the other recorded clinical parameters. These results demonstrate that CD36 expression on monocytes was positively associated with an important and favourable clinical outcome for PAM.

### High Nrf2 and HO-1 mRNA levels correlate with low parasite density and high infant birth weight

**Fig. 3:**
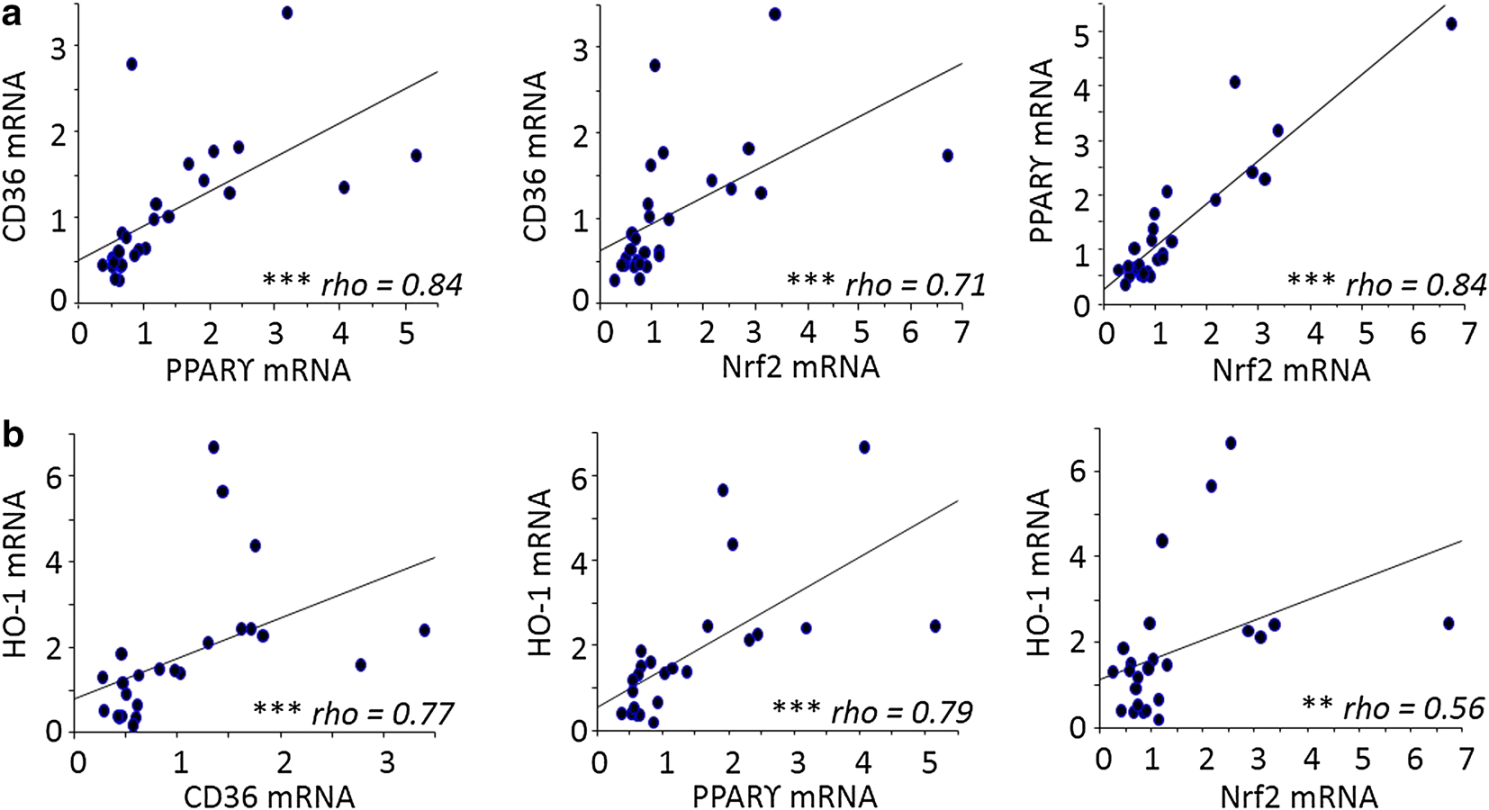
Correlations between CD36, PPARγ, Nrf2 and HO-1 mRNA levels expressed by monocytes sampled in Beninese women presenting malaria at delivery. **a** Correlations between CD36/PPARγ, CD36/Nrf2 and PPARγ/Nrf2. **b** Correlations between HO-1/CD36, HO-1/PPARγ and HO-1/Nrf2. Statistical significance was tested by the Spearman correlation test. ***P < 0.0005, **P < 0.005, *P < 0.5

**Fig. 4:**
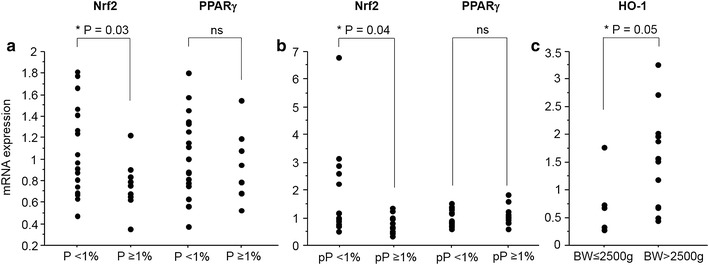
Influence of Nrf2, PPARγ and HO-1 mRNA levels on clinical parameters in PAM. Correlations between Nrf2 or PPARγmRNA levels were related to peripheral (**a**) and placental parasitaemia (**b**). **c** HO-1 mRNA level correlation to birth weight. Values were compared by the Mann–Whitney U-test

To further the analysis carried out on CD36, the study focused on the different transcription factors regulating CD36 expression on monocytes. The transcriptional regulation of CD36 is known to involve both the PPARγ and Nrf2 transcription factors [24, 25]. HO-1 mRNA levels were also examined, as its gene regulation directly depends upon Nrf2 activation. CD36, PPAR-γ, Nrf2 and HO-1 mRNA levels were all highly correlated with each other. Specifically, CD36 and PPAR-γ were strongly correlated with each other and Nrf2 and HO-1 [P < 0.0001 by the Spearman test, correlation coefficient (rho) = 0.71–0.84]. Nrf2 and HO-1 were similarly well correlated (P < 0.001, rho = 0.56) (Fig. 3). The potential link between these factors and various clinical features associated with PAM was investigated. Nrf2 mRNA levels were higher in monocytes from pregnant women presenting peripheral or placental parasitaemia <1 % (Fig. 4a, b). Additionally, higher levels of HO-1 mRNA were linked with increased infant birth weight (Fig. 4c). No clinical features were found to be associated with PPARγ (see Fig. 4a, b). These results outline the potential beneficial role of the Nrf2/HO-1/CD36 axis on PAM clinical outcomes.

### Nrf2 activation can restore CD36 levels in monocytes from mothers with low birth weight children

**Fig. 5:**
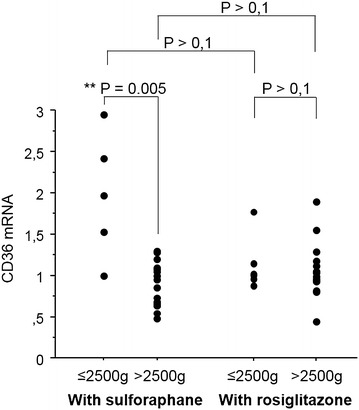
Capacity of monocytes to modulate CD36 expression in PAM. Monocytes were treated with sulforaphane (10 µM) and rosiglitazone (5 µM), two respective agonists of Nrf2 and PPARγ for 4 h before the detection of CD36 mRNA level by qRT-PCR. *Left panel* Monocytes sampled in women with low infant birth weights (≤2.5 kg) produced higher levels of CD36 mRNA after sulforaphane activation than monocytes related to high infant birth weight (>2.5 kg). *Right panel* Activation of these two groups of monocytes with rosiglitazone did not lead to any difference. Monocytes activated by sulforaphane were also compared to monocytes activated by rosiglitazone. No difference of capacity to produce CD36 mRNA was found, whatever the group of monocytes. Values were compared by the Mann–Whitney U-test

To assess the capacity of monocytes from PAM patients to modulate CD36, the mRNA level of CD36 upon rosiglitazone or sulforaphane treatment, two respective PPAR-γ and Nrf2 inducers, was measured. Furthermore, the level of gene induction was also compared between mothers who bore infants with low (MoLBW) and high (MoHBW) birth weights. Interestingly, MoLBW displayed significant increases in CD36 transcription following sulforaphane treatment compared to MoHBW (Fig. 5, Mann–Whitney U-test, P = 0.005). It is important to note here that the maximum level of CD36 mRNA did not differ between the two groups, and that the increase was due to a low basal level of CD36 in the MoLBW. Rosiglitazone treatment failed to induce CD36 mRNA induction in both groups of women.

### CD36, Nrf2, PPARγ and HO-1 mRNA levels were higly corelated to anti-inflammatory markers IL-10 and CD163

**Fig. 6:**
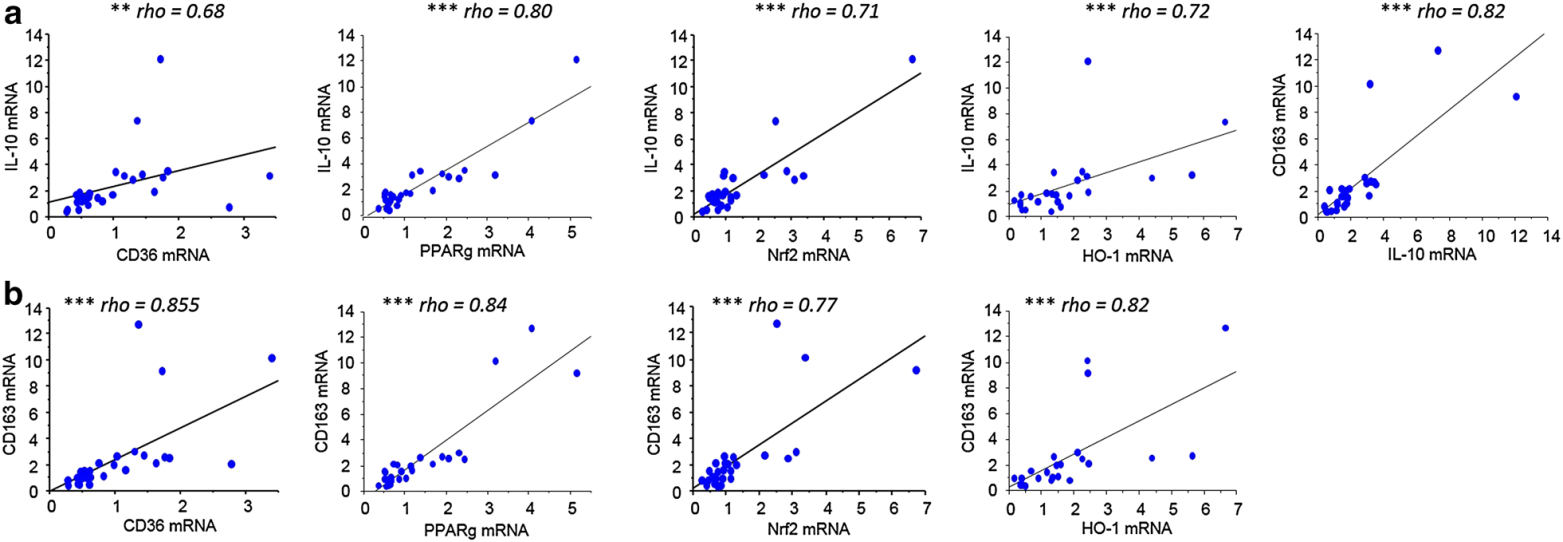
Correlations between CD36, PPARγ, Nrf2, HO-1 mRNA levels and IL-10 and CD163 mRNA levels expressed by monocytes sampled in Beninese women presenting malaria at delivery. **a** Correlations to IL-10. **b** Correlations to CD163. Statistical significance was tested by the Spearman correlation test. ***P < 0.0005, **P < 0.005, *P < 0.5

The relationship between anti-inflammatory markers and CD36 expression was examined, as well as PPARγ, Nrf2 and HO-1. As shown in Fig. 6, CD36, PPARγ, Nrf2 and HO-1 mRNA levels were all positively correlated with IL-10 (Fig. 6a) and CD163 mRNA levels (Fig. 6b) (Spearman correlation test, P < 0.005 to P < 0.0001). CD163 and IL-10 were also highly related (Fig. 6a).

## Discussion

Monocyte/macrophage accumulation in placental blood intervillous spaces during PAM is widely associated with poor clinical outcome [4–6]. The objective of this study was to examine the role of the monocyte/macrophage receptor CD36 and the detoxifying enzyme HO-1 during PAM. In this study, monocytes from Beninese pregnant women infected with malaria at delivery were studied. A total of 27 pregnant women were included in the study and subdivided into two groups based on the birth weight of their infants. For the first time, a correlation between Nrf2-driven CD36 and HO-1 gene expression on circulating monocytes and a positive outcome during PAM was demonstrated on clinically relevant samples. While the low number of MoLBW (n = 5) is a limitation, the use of multiple and complementary statistical tests helps to validate and strengthen the findings. More statistically powered studies will be needed to explore possible mechanisms.

Low infant birth weight constitutes one of the main adverse outcome for PAM, due to restricted growth during pregnancy and to placental circulation disturbances [39], for which there are two non-excluding hypotheses. One is based on impaired uterine artery remodeling which leads to the release of pro-inflammatory cytokines that cause hemodynamic disturbances in the utero placental circulation [40]. The other is based on mechanical obstruction of the intervillous spaces due to infected erythrocytes and monocytes, and fibrin deposition [41]. The present report supports the second hypothesis, as the involvement of CD36 implicates the elimination of *Pf*-iE and apoptotic cells which would likely contribute to the removal of any obstructions in the intervillous spaces. However, the CD36 adhesion phenotype is not a common feature of malaria, as placenta-sequestered parasites are known to more strongly adhere to CSA as opposed to CD36 [22, 42]. CSA-binding isolates were indeed shown to present a defect for non opsonic phagocytosis in vitro [43]. Importantly, placental pathology during malaria infection in mice has been reported to depend on a mitochondrial-dependent apoptotic pathway occurring through increased lipid peroxidation thus jeopardizing the materno–fetal relationship [44]. Considering that CD36 plays an important role in the non-inflammatory elimination of apoptotic cells [14], it is tempting to speculate that CD36 may also regulate the elimination of dead cells at the placental level during PAM, which would maintain feto–maternal exchanges and increase birth weight at delivery.

The other known function of CD36 during malaria infection includes its collaboration with TLR2 to trigger inflammatory response to *Pf*-GPI anchors [19]. This should logically not correlate to higher birth weights as the inflammatory response in the placenta is thought to lead to low birth weight by damages caused to the placenta villi and the disturbance of feto–maternal exchanges [6, 45]. In this study, birth weight was not related to cytokine levels, but an increase in IL-10 plasma levels was noticed in response to increased parasite densities which is in line with previous literature proposing IL-10 as a biomarker of *P. falciparum* infection during pregnancy [46]. Production of IL-10 during PAM may help in counteracting the consecutive damages of the inflammatory response. In a longitudinal study comparing uninfected and *P. falciparum* infected women during pregnancy, the increase of IL-10 level during PAM was combined to increased IP-10 level and decreased RANTES level regardless of gestational age at the time of infection [47]. RANTES, a chemokine implied in leucocyte recruitment at inflammatory sites, has been associated with severity and mortality in malaria children [48, 49]. In addition, RANTES production was shown to be IL-10-dependent in vitro [50]. IP-10 has also been identified as a biomarker for mortality in children with cerebral malaria [51] or severe malaria [52]. IL-10, IP-10 and RANTES are thus probably implied in the control of the pro- and anti-inflammatory balance, a key factor during PAM for limiting both infection and foetus damages.

Interestingly the CD36 scavenger receptor was strictly expressed by CD14^+^ CD16^−^ and CD14^+^ CD16^+^ monocytes, and its expression was extremely low in CD14^low^ CD16^+^ monocytes. Intermediate monocytes (CD14^+^ CD16^+^) are thought to be largely anti-inflammatory by producing high levels of IL-10 and increasing phagocytic activity [53]. The overexpression of CD36 on these cells would contribute to the former, however CD36-dependent phagocytosis may not be very relevant during PAM. On the other hand, CD36 and IL-10 mRNA levels in monocytes from this cohort were positively correlated, suggesting that CD36 expression was involved in an anti-inflammatory process. The anti-inflammatory response during malaria also implies the receptor CD163 which scavenges the complex haemoglobin:haptoglobin, allows heme degradation by HO-1, avoids tissue damage [54, 55], and facilitates IL-10 secretion [56].

The present work demonstrates a link between the transcription of CD36, CD163, HO-1 and IL-10 mRNA during PAM. CD36, Nrf2 and PPARγ were also related to CD163 expression, reinforcing the anti-inflammatory aspect of CD36 expression on monocytes. Chua et al. reported an association between the soluble form of CD163 (sCD163) and poor clinical outcomes during PAM, i.e. lower hemoglobin levels and lower infant birth weights compared to uninfected pregnant women [57]. sCD163 is the result of CD163 shedding induced by TNF [36]. sCD163 levels were shown to be inversely correlated to membrane-bound CD163, suggesting that high sCD163 levels may reflect lower membrane-bound CD163 activity [58]. These results combined with the present study suggest that a greater activity of membrane-bound CD163, or a higher expression of CD163 on the cell surface, would promote favorable clinical outcomes during PAM by limiting inflammation due to free hemoglobin and heme. This possibility, however, deserves further investigation.

Non-classical (CD14^low^ CD16^+^) monocytes are described as the pro-inflammatory monocytes producing TNF and little or no IL-10 [59]. Interestingly, a low CD36 expression level was observed in these cells, which correlates with previous findings demonstrating that inflammatory processes, including TNF stimulation, dampens CD36 expression through an inhibition of PPARγ transcriptional activity [29]. These results support the notion that a CD14^+^ phenotype, tolerant to CD36 expression, are linked to beneficial outcomes during PAM. It can be also noted that CD14+ monocytes were largely predominant (80.3 %), despite a noteworthy increase of the non-classical monocyte population compared to healthy pregnancy (see Fig. 1b). CD36 expression may thus have a real impact even if its expression may be lowered compared to uninfected women. However, additional studies could help in understanding the sequence and interdependence of events such as Nrf2 activation, CD36 expression on monocytes, HO-1 and CD163 induction, and IL-10 production during PAM.

PPAR-γ and Nrf2 transcription factors were both shown to regulate CD36 protein expression on monocytes [24, 25]. Olagnier et al. demonstrated that in inflammatory conditions the stimulation of the Nrf2 pathway improved the outcome of severe malaria in mice, whereas PPARγ activation was only slightly active and much less efficient in inducing CD36 expression [29]. In addition, a previous report showed that circulating monocytes from malaria-infected patients presented decreased CD36 protein expression [60]. In this study, the capacity of circulating monocytes from PAM patients to modulate CD36 expression upon rosiglitazone or sulforaphane activation, two respective PPAR-γ and Nrf2 agonists, was evaluated. Only sulforaphane activation induced monocytes from MoLBW to increase CD36 mRNA levels to those of MoHBW. This indicates that MoLBW do not have an impaired Nrf2 pathway, but likely lacked the appropriate stimulation in vivo. Nrf2 activators may be endogenous, particularly the products of oxidative stress (oxygen reactive species, lipid mediators), or exogenous, such as electrophile molecules (curcumin and sulforaphane for example). This result underlines the potential role of the Nrf2 pathway in limiting malaria severity during PAM.

Interestingly, this study showed that increased Nrf2 mRNA levels were associated with lower peripheral and placental parasitaemia during PAM, whereas no such relation was found for PPAR-γ mRNA levels. Thus, it can be postulated that in this Beninese PAM context, the Nrf2 pathway, and not the PPAR-γ pathway, is associated with limited parasite survival and proliferation. Nrf2 is a master regulator of the transcription of many antioxidant and detoxification enzymes, which are required to control the inflammatory process initiated by the detection and phagocytosis of malaria [30]. One such enzyme is HO-1 (encoded by the *Hmox*-*1* gene), known for its role in heme detoxification during malaria by the catabolization of free heme into biliverdin, iron and carbon monoxide [61]. HO-1 was shown to have protective properties in an experimental cerebral malaria model [32, 33]. In this manuscript, a correlation between high HO-1 mRNA levels in monocytes from MoHBW was established, confirming a beneficial role during PAM outcomes. However, recent data propose that high placental parasitaemia leads to an excessive activity of HO-1; intracellular iron accumulation and overload in trophoblasts, inducing cell death and impairing fetal development [62]. Altogether, this suggests that Nrf2 plays a central role in defining the balance between pro- and anti-oxidant mechanisms that appears essential for both infection elimination and host protection.

## Conclusions

This work demonstrates the role of the CD36 scavenger receptor, the enzyme HO-1 and the anti-oxidant pathway Nrf2 in protection against the pathology of malaria during pregnancy. Whereas the antioxidant and detoxifying effects of Nrf2-dependent HO-1 corroborate with other published data, the function of CD36 expression on monocytes during PAM needs further clarification. The positive association of CD36 in PAM clinical outcomes deserves corroboration in other populations and studies, including a larger group of mothers with low birth weight infants.

## Additional file

**Additional file 1: Table S1**. Human primer sequences used in quantitative PCR experiments.
