# Supplementary material for: Nrf2-driven CD36 and HO-1 gene expression in circulating monocytes correlates with favourable clinical outcome in pregnancy-associated malaria
Source: Malar J. 2015 Sep 18;14:358. doi: 10.1186/s12936-015-0888-8 (PMC4575452; doi:10.1186/s12936-015-0888-8)
Supplement: Supplementary file 1 — Additional file 1: Table S1. Human primer sequences used in quantitative PCR experiments. [file 12936_2015_888_MOESM1_ESM.docx]

**Additional Table 1 Human primer sequences used in quantitative PCR experiments**

| **Genes** | **Sequences** |
| --- | --- |
| *CD36* | sense 5' TGT AAC CCA GGA CGC TGA GG  antisense 5' GAA GGT TCG AAG ATG GCA CC |
| *PPAR_ϒ_* | sense 5' GCT GTG CAG GAG ATC ACA GA  antisense 5' GGG CTC CAT AAA GTC ACC AA |
| *Nrf2* | sense 5' GCG ACG GAA AGA GTA TGA GC  antisense 5' GTT GGC AGA TCC ACT GGT TT |
| *Hmox-1* | sense 5' TCC GAT GGG TCC TTA CAC TC  antisense 5' CAA GGA AGC CAG CCA AGA GA |
| *Human 18S* | sense 5' CCT CCA ATG GAT CCT CGT TA  antisense 5' AAA CGG CTA CCACAT CCA AG |
